# Networks for proper versus common name retrieval: insights from causal mapping and new hypotheses

**DOI:** 10.1093/braincomms/fcaf256

**Published:** 2025-06-25

**Authors:** Eléonor Burkhardt, Hugues Duffau, Guillaume Herbet

**Affiliations:** Praxiling Laboratory, UMR 5267, CNRS, University Paul Valéry Montpellier 3, Montpellier 34090, France; Gui de Chauliac Hospital, Montpellier University Medical Centre, Montpellier 34295, France; Gui de Chauliac Hospital, Montpellier University Medical Centre, Montpellier 34295, France; CNRS, INSERM, Institute of Functional Genomics, University of Montpellier, Montpellier 34000, France; Praxiling Laboratory, UMR 5267, CNRS, University Paul Valéry Montpellier 3, Montpellier 34090, France; Gui de Chauliac Hospital, Montpellier University Medical Centre, Montpellier 34295, France; Department of Medicine, University of Montpellier, Montpellier 34090, France; Institut Universitaire de France, Paris, France

**Keywords:** anomia, lexical retrieval, anterior temporal lobe, basal temporal language area, inferior longitudinal fasciculus

## Abstract

Over the years, the anatomical correlates of common name and proper name retrieval have been studied and discussed side by side in the field of cognitive neuroscience. Due to the particular semantic status of proper names (i.e. meaningless marker referring to unique entities with a token link), it is generally acknowledged that their lexical retrieval is cognitively more demanding than that of common names. Thus, the neural networks engaged for accessing the phonological output lexicon of common names and proper names could be partially segregated, centred on different cortical regions and underpinned by distinct structural pathways. However, this possible anatomo-functional organization has not yet been explored in an in-depth review. Hence, the objective of our review is 3-fold: (i) to highlight the cortical networks underlying common name and proper name retrieval; (ii) to summarize the findings from recent studies using causal methods of tract-oriented functional mapping (i.e. direct electrostimulation and disconnection-symptom mapping) with the aim of isolating ventral structural connections that contribute to common name and/or proper name retrieval; (iii) to develop hypotheses regarding the anatomical connectivity implicated in lexical access to common names and proper names, in relation to the input modality and semantic category (for proper names, in the latter case). Our critical review first suggests that, although both categories appear to rely on a widely distributed and overlapping left fronto-temporo-parietal cortical network, the mid-to-posterior part of the ventral temporal cortex (especially the fusiform gyrus) and the anterior temporal structures (including the temporal pole), seem more specifically involved in common name retrieval and in proper name retrieval respectively, regardless of their input modality. Second, the left inferior longitudinal fasciculus appears to play a crucial role in picture-based lexical retrieval for both common names and proper names. Based on these key findings, we propose tentative models of anatomical connections underlying lexical retrieval. We suggest that distinct components of the inferior longitudinal fasciculus are implicated in lexical retrieval for common names and proper names and that another similar segregation mediates the retrieval of face names and landmark names. We further hypothesize that the left middle longitudinal fasciculus plays a role in auditory-guided lexical retrieval, owing to its projections into the anterior temporal structures and its close relationship with the auditory associative cortex. Within the framework of the dual-language model, we discuss the heuristic value of these anatomical hypotheses to understand the mechanisms by which anomia arises in various neurological conditions, providing insights and directions for future studies.

## Teaser

Burkhardt *et al*. review the neural systems involved in common versus proper name retrieval, highlighting the left ILF as a critical white matter tract and discussing new linguistically constrained anatomical hypotheses that consider grammatical and semantic categories, as well as input modality.

## Graphical Abstract

**Graphical Abstract:**
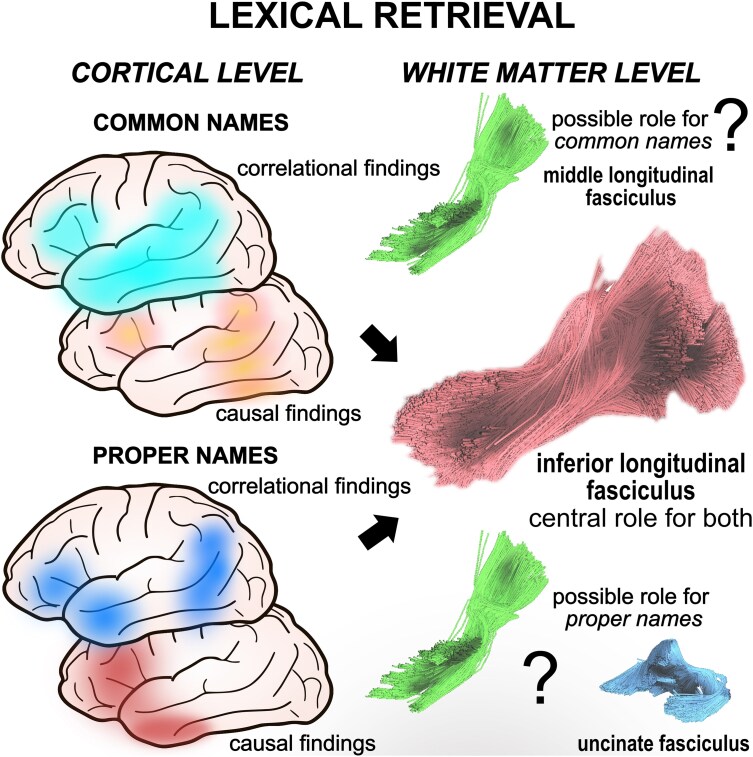

## Introduction

For many years, it was commonly believed that aphasia syndromes resulted from lesions affecting discrete and highly specialized areas of the cerebral cortex. An iconic example of this belief is the association between Broca’s area and the impairment of speech production, as described by Paul Broca in 1861.^1^ However, it gradually became evident that localizationist or simple associationist models could not fully account for both the neural organization of language and the complexity of aphasic disorders.^2,3^ Decades of neuropsychological studies have consistently shown that neuropsychological syndromes, in particular aphasia, stem from the disruption of brain-wide neural networks formed by distributed cortical and white matter structures.^4-9^ In the realm of language processing, contemporary network perspectives underscore the fundamental role of a predominantly left lateralized, interactive dual-stream neural system. Within this intricate neurobiological framework, the dorsal pathway mediates the precise execution of phonological and articulatory processes, while the ventral pathway orchestrates the complex mapping of percepts onto semantics.^10-12^

From a neuroanatomical standpoint, an accumulating growing body of empirical evidence substantiates the pivotal roles played by the arcuate fasciculus and the superior longitudinal fasciculus within the dorsal stream. These white matter tracts constitute the foundational white matter architecture through which phonological and speech articulatory information is seamlessly integrated. In a parallel manner, the inferior fronto-occipital fasciculus (IFOF) (and the so-called extreme capsule) establish critical connections within the ventral stream for verbal and non-verbal semantic processing.^6,13-15^ Furthermore, the complex tapestry of language processes also encompasses the participation of the inferior and middle longitudinal fasciculi (ILF and MdLF), in addition to the uncinate fasciculus (UF), as parts of the ventral stream. It is noteworthy that the precise functional role of these tracts within the domain of language remains a subject of ongoing scientific exploration and this review endeavours to shed light on this particular aspect.

In the comprehensive study of the language network, the deployment of tract-oriented lesion-symptom mapping approaches offers a valuable means of exploring the relationship between surgery-induced disconnections and cognitive impairment. They are especially relevant in neurosurgical glioma patients since the anatomical connections serve as the primary conduits for tumour spreading and are generally less amenable to functional compensation, compared to associative cortical areas.^16,17^ In this context, these methods offer the advantage of identifying anatomo-functional associations, even in the presence of neuroplasticity.^18^ Thus, the disconnection-symptom mapping in patients with glioma has unveiled significant new insights regarding the language ventral stream.^14^ However, it is worth noting that some of these findings, especially those related to lexical retrieval, despite their importance, have yet to be comprehensively discussed and fully incorporated into existing network models of language.

In this review, we are interested in the neural networks responsible for lexical access to common names (CNs) and proper names (PNs), with a specific emphasis on ventral white matter connectivity. From a cognitive standpoint, naming an object involves a complex set of interacting processes that encompass perception, conceptual processing, word selection, phonological form retrieval, and, ultimately, verbal production through articulatory planning and execution.^19-21^ It also implicates supra-linguistic cognitive control functions. As a result, brain damage can disrupt naming abilities in various ways, depending on the specific process affected.^22,23^ In this neuropsychological context, pure anomia is commonly described as arising from a disconnection between preserved semantic knowledge and an intact phonological output lexicon.^24^

On the other hand, while CNs (e.g. ‘blueberry’ or ‘table’) are meaningful and category-related, PNs (e.g. ‘John’ or ‘Geneva’) are meaningless and individuality-related.^25,26^ Thus, whereas the former are generally substitutable (with circumlocutions/synonyms), the latter are not,^27^ which is particularly significant in the context of aphasia. Furthermore, PNs, especially when associated with faces, might involve peripheral socio-emotional processing^28,29^ and their lexical access may place a greater demand on memory resources compared to CNs, particularly in terms of episodic processes.^25,26^

Consequently, it has been hypothesized that the neural systems responsible for lexical access to PNs and CNs partly differ^25,26^ although this issue remains unresolved to date. In this context, some authors argue for a single semantic system (notably including lexical-semantic aspects), whose disruption may lead to naming impairments affecting both grammatical categories.^30^ However, cognitive dissociations observed in neurological patients between anomia for CNs and PNs also provide some interesting insights. While there are a few reported cases of patients showing anomia for CNs without anomia for PNs,^31-34^ albeit under particular circumstances (e.g. sparing of country names only, deficit at the semantic level, or deficit confined to the oral modality, etc.),^26^ the reverse pattern, especially for person names, is more frequently reported.^32,35-39^ Therefore, it is somewhat expected that circumscribed damage to specialized cortical or subcortical structures can occasionally lead to selective anomia, especially for PNs and to a lesser extent, for CNs.

It is worth noting that previous reviews have thoroughly explored the cognitive (neuro)psychology of PNs, sometimes in relation to their potential neural underpinnings, compared to CNs.^25-27,40,41^ However, none has attempted to dissociate the neural networks involved in the lexical access to these two grammatical categories, especially at the white matter level. This gap exists due to the only recent shift in research focus towards specifically exploring the role of ventral structural connectivity in lexical access.^42-48^ In this context, the primary aim of this critical review is to disentangle the different contributions of the ventral neural circuits to lexical retrieval, placing a particular emphasis on the ventral white matter tracts, while acknowledging the relatively limited availability of data related to the latter. Crucially, highlighting white matter damage in the context of anomia may help clarify the relationships between patients’ aphasic impairments and lesion topography, thereby advancing our understanding of the network-level anatomo-clinical mapping of naming deficits.

In the opening section of this review, we offer a comprehensive update on the cortical foundations of CN and PN retrieval, with a special focus on identifying commonalities and distinctions within the cortical systems underlying these processes. In the next section, we delve into the various anatomical connectivities implicated in CN and PN retrieval, with a special emphasis to the left ILF, for which causal evidence has accruing until recently.^42-45,48^ Finally, in the last part of this review, we present hypothetical network models for PN/CN retrieval. These models serve as a foundational framework for developing new linguistically constrained hypotheses regarding the axonal connections involved in retrieval processes, in relation to the grammatical class of names (PNs and/or CNs), semantic subcategories within PNs (famous persons/famous landmarks) and the input modality, for both CNs and PNs (visual versus auditory stimuli).

## Cortical networks for proper name and common name retrieval: an update

As a pre-requisite, it is important to clarify that neuroscience and neuropsychological studies have generally not strictly compared the cortical bases involved in PN versus CN retrieval. While one cortical mapping study^49^ administered famous face and object naming tasks to patients with brain tumours during awake surgery and highlighted a number of dissociated functional sites, it did not investigate the critical basal temporal structures. Other research has mainly focused on CN and PN retrieval separately, often using picture naming tasks^22,50,51^ or famous face naming (FFN) tasks^52,53^ respectively, while controlling for several cognitive processes (e.g. recognition/access to semantic information for unique entities,^54^ and recognition or fluency for non-unique entities^50^). Alternatively, some studies have investigated the effect of semantic categories on naming, including equally both common (animate and inanimate objects) and unique entities (mainly faces).^28,55^ Lastly, a clinical group study^30^ and numerous case studies^26^ investigated CN and PN retrieval using comprehensive language assessments but the former only concerned patients with ATS damage while the latter did not included anatomo-clinical analyses. Of note, ATS generally correspond to the anterior aspects of all temporal gyri (including the temporal pole, the superior, middle and inferior temporal gyri together with the fusiform gyrus).^56^

Regarding PNs specifically, a set of behaviour-based neuropsychological^57-59^ or neuromodulation^60^ studies focusing on ATS, analysed the modality effect (verbal/non-verbal, mainly corresponding to naming/recognition) on performance, depending on the hemisphere laterality. Other works investigated the sensibility of the ATS to the input modality in people naming tasks, depending on whether they were presented with their voice or with their face.^60,61^ In the same way, some studies were interested in the role of the ATS in naming unique entities depending on the semantic category (e.g. landmarks versus famous faces).^52,62^ Importantly, our review excludes findings related to associative face/name learning^63^ and instead focuses on lexical retrieval of PNs associated with well-known unique entities, typically assessed using overt naming paradigms (as well as of non-unique entities, generally assessed with naming tasks). Particular attention was given to distinguishing causal findings from correlational results.

## Common names

### A large-scale distributed network

Numerous studies have sought to elucidate which brain areas are responsible for concept-to-word mapping, using various methodological approaches. These includes functional^22,28,64-68^ and structural^69-72^ neuroimaging, lesion-symptom mapping^44,50,51,73-75^ and electrical stimulation,^65,76^  ^,77^ all of them involving diverse patient populations (such as those with neurodegenerative diseases, epilepsy/temporal lobectomy, tumours or strokes) and healthy subjects.^28,66,78^ More marginally, a few behavioural studies, notably conducted with patients affected by posterior cortical atrophy, have also provided relatively valuable insights.^79-81^

**Figure 1:**
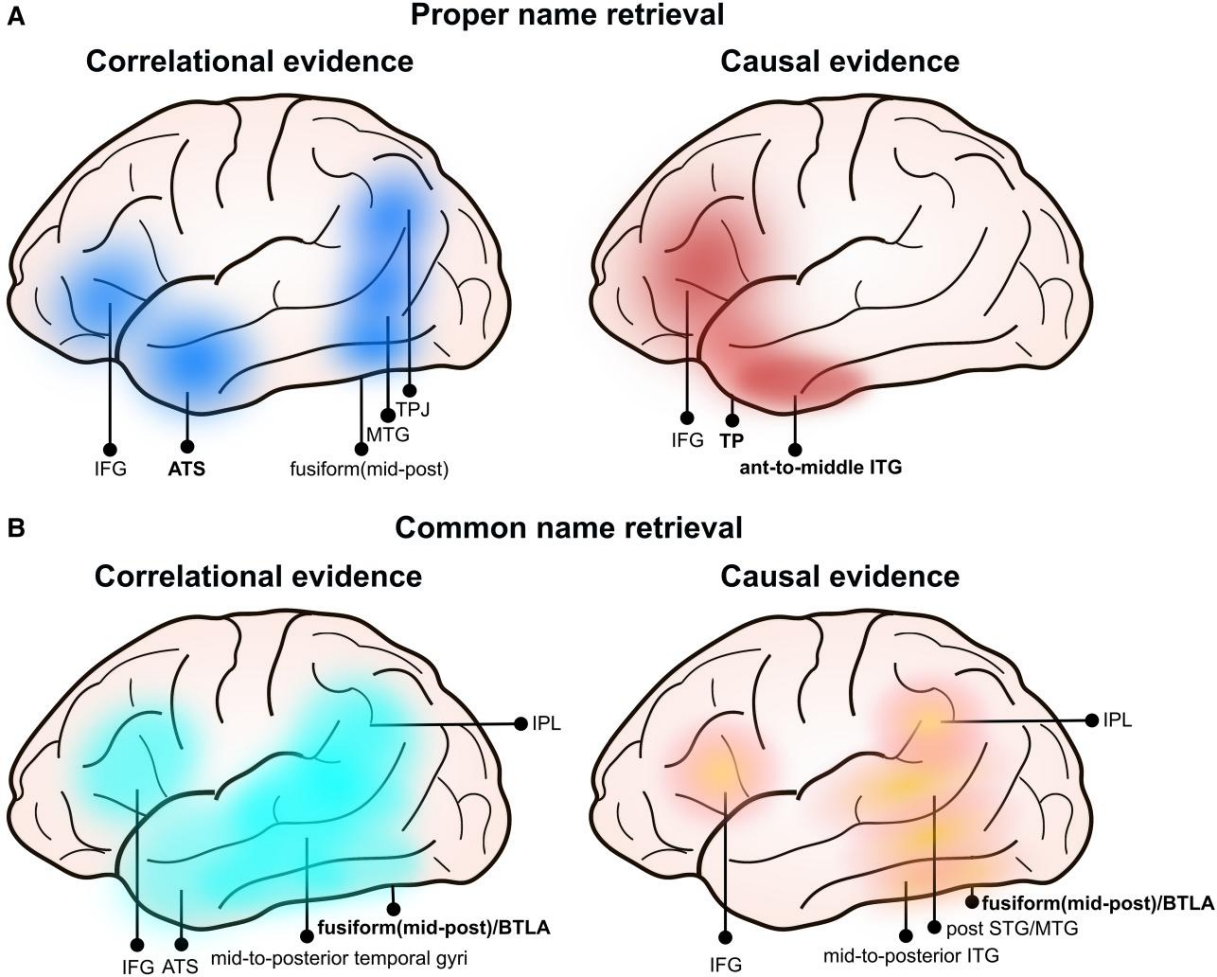
**The cortical network implicated in lexical access to (A) PNs and (B) CNs.** The regions reported are those the most commonly identified in studies conducted in both patients and healthy subjects. Causal evidence (right panel) is differentiated from correlational evidence (left panel), highlighting the potential greatest implication of anterior versus posterior temporal regions in PN and CN retrieval, respectively. The figure does not include exhaustive information on medial areas potentially significant for PN retrieval, which encompass the medial section of fronto-temporo-parietal regions including the orbitofrontal regions,^28,29^ the anterior cingulate cortex,^28,29,92^ the anterior parahippocampal gyrus^28^ and the posterior cingulate/retrosplenial cortex.^92^ The functional central hubs are indicated in bold. ATS, anterior temporal structures; BTLA, basal temporal language area; IFG, inferior frontal gyrus; IPL, inferior parietal lobe; MTG, middle temporal gyrus; STG, superior temporal gyrus; TP, temporal pole; TPJ, temporo-parietal junction.

The generated findings have indicated that lexical retrieval/lexical production predominantly engages a leftward cortical network, primarily located along the ventral language stream. This network encompasses key temporal structures, particularly the inferolateral and ventro-basal regions,^22,44,51,64,67,70,72,77,82,83^ which mostly concern their mid-to-posterior parts, occasionally involving the occipito-temporal areas.^28,74,77^ It also includes the posterior superior^67,72,76^ and middle temporal areas,^50^ further extending into temporo-parietal/inferior parietal regions.^22,65,67,75,82^ Additionally, it involves the frontal operculum^22,65,66,73,78,82^ and, in some cases, parts of the middle frontal gyrus.^66,84,85^ Significantly, this widely distributed fronto-temporo-parietal cortical network is robustly supported by findings from direct electrical stimulation (DES) studies conducted intraoperatively using a picture naming task,^84-86^ which particularly underscore the causal role of the posterior part of the superior temporal gyrus. Moreover, the importance of the hippocampus in visually guided naming following anterior temporal lobectomy has been highlighted in patients with intractable epilepsy, provided that the hippocampus is not sclerotic before the surgery.^87-90^Consistently, meta-analyses of functional neuroimaging studies^91^ and of lesion-symptom mapping investigations^75^ generally align with these studies, placing an emphasis on the mid-to-posterior segments of the temporal lobe (see Fig. 1).

### Central temporal cortical areas

#### The mid-to-posterior ventral temporal regions

The region commonly known as the ‘basal temporal language area’ (BTLA), whose the location is mainly assigned to the mid-to-posterior part of the fusiform (corresponding to Brodmann’s area [BA] 37),^51,93,94^ serves as a central hub within the cortical network responsible for integrating semantic features with lexical labels. Pioneering electrostimulation studies^95,96^ paved the way for subsequent research, with recent studies consistently confirming that disruption of the BTLA leads to anomia. This has been evidenced through other inhibitory cortical stimulation^65^ (see also^77^) together with voxel-based lesion-symptom mapping (VLSM) studies related to surgical resection procedures.^44,51,83,93^ Functional neuroimaging studies have further associated naming deficits with tissue dysfunction in this area^22,94^ (see also^65^). Furthermore, Hillis *et al*.^23^ demonstrated that a few days after a stroke, the restoration of blood flow in the left BA37 was linked to the recovery of lexical access.

#### The anterior temporal structures

Moreover, functional neuroimaging,^28,68^ lesion mapping^28,54,68^ and behavioural^59,97,98^ studies, sometimes combined with structural/morphometric MRI analyses^99-102^ have repeatedly linked the left anterior temporal structures (ATS) with semantically driven lexical access (sometimes for a given semantic category), in various patient populations. However, naming failures resulting from ATS damage seem more frequently associated with semantic disturbances, at least evident in language production.^73,99,103-107^ As such, the issue of the ATS’s precise involvement in lexical retrieval remains steeped in controversy, particularly within the framework of the ‘hub-and-spoke’ model (see also^51,108^), which preferentially emphasizes the role of bilateral ATS in modality-independent semantics,^109,110^ including in the context of cortical stimulations.^111^

### Synthesis

Taken together, a range of multi-modal studies has demonstrated that lexical retrieval of CNs primarily depends on the left ventral language pathway (including fronto-temporo-parietal regions), within which temporal regions play a critical role, especially with regard to the mid-to-posterior part of the ventro-basal temporal cortex and of the inferolateral one to a lesser extent (i.e. the BTLA), but also to the ATS, albeit in a likely less specifically way.

## Proper names

### A large-scale distributed network

Large scale lesion-deficit mapping study, mostly based on cerebrovascular patients, and supplemented with functional neuroimaging,^28,68^ have provided compelling evidence that the retrieval of PNs depends on a mainly left lateralized neural network. In particular, Damasio *et al*.^28^ identified the temporal pole (TP) and the neighbouring anterior inferior temporal and anterior parahippocampal cortex, as well as the ‘pars orbitalis’ of the inferior frontal gyrus, as critical structures for PN retrieval. The anterior cingulate gyrus and the orbitofrontal areas may also contribute.^28,29^ The involvement of fronto-temporal regions has received further support from DES studies^49^ and PET/functional MRI (fMRI) research involving healthy subjects.^62^ Generally, the neural network of PN retrieval encompasses both medial and lateral fronto-temporal regions^28,29,49,62,112^ and, occasionally, extends into the posterior middle temporal cortex and the temporo-parietal junction (see Fig. 1).^113^

### Central temporal cortical areas

#### The key role of the anterior temporal structures in lexical-semantic processing of unique entities

Within this extensive neural network, specific sectors of the left ATS, including the TP, serve as central hubs for retrieving the names of famous individuals from faces.^43^ According to Damasio *et al*.’s theory,^28,114^ the ATS function as a ‘convergence zone’ for processing unique entities. This implies that they may index inputs originating from modality-dependent regions and establish connections in a bidirectional manner, with verbal labels referring to known unique entities (involving both the mapping from knowledge of unique entities to their names, and the retrieval of knowledge about these entities from their names^115^).

Overall, the ATS involvement in lexical retrieval, especially for PNs, finds robust support in an array of neuropsychological studies associated with fMRI/PET investigations.^28,29,52,53,68,114^ These findings have been consistently substantiated by other advanced lesion-symptom mapping studies^43,54^ and by voxel-based morphometry^116^ together with neuromodulation^60,117^ and electrophysiological works.^61^ They are also echoed in neuropsychological behavioural research, frequently conducted in patients with epilepsy/who underwent surgery to treat drug-resistant temporal epilepsy.^52,57-59,118^ Overall, the role of the left-biased ATS includes lexical access for various categories such as famous faces,^28,42,43,60,116,118^ landmarks,^62,114^ speaking voices^60,61,119^ and famous melodies with or without lyrics.^112,120^

#### Challenging the specific role of the anterior temporal structures in unique entities processing

The ‘hub-and-spoke’ model challenges the understanding of the ATS's role in the processing of unique entities and PN retrieval, as particularly driven by specific semantic or grammatical categories. No special emphasis is neither placed on the left ATS’s role in lexical access. Indeed, this theory rather assumes that the ATS involve bilaterally high-level semantic integration regardless of the process involved, encompassing all classes of objects. In this alternative framework, the ATS constitute a graded transmodal representational system^56^  ^,109,110,121^ enabling conceptualization process, by establishing functional connections with modality-dependent association areas.^122^ Furthermore, the ATS, especially its ventrolateral part/the anterior fusiform gyrus, is not merely conceived as a binding zone. Rather, it is suggested to support, through experience, the building of nonlinear transmodal and coherent generalizable conceptual knowledge.^109,110^ Moreover, this framework assumes that the potential functional differences between verbal and non-verbal semantically related processes, associated with the left versus the right ATS, are not attributable to a lateral specialization of the ATS *per se*.^56,110^

It holds true that injury to the left ATS typically results in anomia without significant semantic impairment, as evidenced by various neuropsychological studies.^30,108,123^ In line with this, other works conducted in healthy subjects demonstrated that transcranial magnetic stimulation (TMS)-related virtual lesioning of the left ATS increased naming latencies.^122,124,125^ Nonetheless, this pattern is not understood as linked to the ATS’s hemispheric specialization but interpreted in the light of two main factors: the bilateral organization of the ATS, which confers increased resilience to unilateral neurological damage for semantic processing and an asymmetric connectivity strength leading to a leftward hemispheric linguistic bias.^109,110,126^ In a similar vein, there may be reciprocally, a lateralized functional connectivity for nonverbal stimuli, in particular faces, into the right ATS.^127^

#### The hypothesis of the anterior temporal structures’ sensitivity to an effect of high semantic specificity

We have previously discussed the challenges that the ‘hub-and-spoke’ model presents to a theory suggesting that the ATS are sensitive to naming effects, especially based on semantic/grammatical categories. Some researchers have thus rather assumed that the ATS’s involvement may vary, depending on the specificity level of processing demands required by a given lexical-semantic task (i.e. greater implication with highly specific requirements).^30,124,128,129^

This proposition is grounded in few studies, which found an ATS’s implication in tasks involving visual-semantic distinctions at a specific level. As such, significant results were obtained by Rogers *et al*.^128^ in their category verification paradigm, where participants indicated whether the pictorial input matched a conceptual category (corresponding to either animals or vehicles) at three levels of specificity (general, basic and specific). In this PET functional imaging study, the authors found a significant relationship between the ATS activation and categorization at a specific level (e.g. ‘Labrador’), as opposed to an intermediate (e.g. ‘dog’) and general (e.g. ‘animal’) levels, without category and/or domain effects. The hypothesis of the ATS’s sensitivity to a specific effect has also been supported by at least one study^124^ which showed a significant repetitive TMS (rTMS) slowing naming effect in healthy subjects, but only at a specific level. Moreover, neuropsychological evidence derived from semantic dementia^128,129^ further suggested that this neurological condition, which affect the ATS, impairs semantic performance by a characteristic specificity effect. Indeed, Rogers *et al*.^129^ showed that performance in semantic dementia patients and to a lesser extent, in stroke patients, across several semantic tasks (picture naming, picture sorting, word-picture matching and fluency) with high specific demands, was significantly worse than performance assessed at basic or general levels.

Critically, Rogers *et al*.^128^ found that the same sectors of the ATS typically damaged in semantic dementia (impacting their semantic performance with a specificity effect^129^) and implicated by the specific level of classification in healthy subjects, were activated during a FFN task.^55^ Accordingly, the ATS may be involved in processing stimuli from a large array of categories rather than unique entities exclusively, particularly under more stringent conditions, in terms of level of classification. In this sense, the ATS activation responded selectively to famous faces/names, compared to other categories, notably named at a basic level.^55^ This suggests that the unique entities may be distinguished from common objects only to the extent that the latter are generally treated at a basic level, implying a more coarse-grained processing than the former, treated at a specific-level. In this line, a study showed that epileptic patients who had undergone anterior temporal lobectomy exhibited naming impairments for both faces and objects, although for object naming, this impairment tended to be present only when reaction time was measured or when the complexity of the tasks increased (e.g. probing less familiar concepts)^30^ (see also^130^). Accordingly, the ‘hub-and-spoke’ theory posits that both processing of PNs and CNs depend on the same semantic system and are susceptible to be impaired in case of neurological damage to the ATS. However, due to the finer distinctions required by the former, lexical-semantic processing might be prone to deficit more salient for PNs than for CNs.

#### The mid-to-posterior ventral temporal regions

Interestingly, electrophysiological findings have suggested the involvement of the posterior part of the fusiform gyrus in naming famous faces, based on intracranial recordings, in three cases of epileptic patients.^61^ Likewise, a PET study conducted in few healthy subjects has suggested the possible implication of the mid-posterior basal temporal region (BA37) in lexical access for all categories (except colours), including famous faces.^55^ However, it is worth noting that recent VLSM findings conducted in a relatively wide cohort of patients affected by a low-grade glioma, did not show the critical implication of the mid-to-posterior ventral temporal regions in FFN retrieval, contrary to the ATS (including the TP).^43^ Thus, it seems that the posterior ventro-basal temporal regions are generally not essential for PN retrieval (at least for faces) though they may have a contributory role. Finally, PN retrieval might rely in part on cortical structures already shown to be crucial for CN retrieval, just as some studies have reciprocally suggested an involvement of the ATS in CN retrieval.^101^ However, further direct evidence for this claim is actually lacking.

### Synthesis

The cortical network underpinning PN retrieval mainly comprises left fronto-temporal regions, occasionally extending into mid-posterior ventro-temporal regions and, to a lesser extent, into the temporo-parietal junction. Concerning key temporal regions, the ATS seem represent a critical functional epicenter of this network while the posterior temporal regions likely have a contributory role. However, the specific role of the ATS in processing unique entities, including PN retrieval, has been questioned by the ‘hub-and-spoke’ model.

## The contributing roles of cortical regions beyond the two temporal hubs

Generally, due to the multitude of processes they imply, naming abilities rely on a widespread neural system. As a result, naming failures can arise due to other reasons than lexical access impairment, which primarily involves lesion to temporal structures.^75^ As such, damage to the fronto-parietal regions/the dorsal language stream (mediated by the arcuate fasciculus) can lead to phonological paraphasia (or anomia) whereas damage to the fronto-temporal regions/the ventral semantic stream (mediated by the IFOF), can result in semantic paraphasia (or anomia),^6^ at least for CNs. Otherwise, the disruption of the occipito-temporal network^131^ (including the posterior part of the ILF)^132^ can, in turn, provoke visual recognition impairments, possibly leading to visual paraphasias. Likewise, damage to the neural systems involved in motor speech output, speech initiation and control, especially those connecting the supplementary motor area to the caudate nucleus (via the fronto-striatal tract) or to the posterior inferior frontal gyrus (via the frontal aslant tract), may cause speech dysfluencies during naming^133,134^ and articulatory errors.^22^ Further, damage to the inferior frontal regions are likely to contribute to an executive deficit in selecting information from competing lexical-semantic alternatives.^73,135-138^

In this vein, a PET study conducted in patients with a probable Alzheimer disease aimed to isolate the mechanisms underlying both picture naming and verbal fluency.^139^ This study showed that metabolic activity in inferior frontal regions was associated with naming performance facilitated by phonemic cueing (among other regions), and with phonological fluency abilities (in isolation). In contrast, spontaneous naming performance was associated with activity in inferior temporal structures, while semantic fluency abilities involved both frontal and temporal regions. The authors thus suggested that naming failures may result from dysfunction in the inferior frontal regions, disrupting controlled retrieval processes. On the other hand, dysfunction of the temporal structures may lead to lexical-semantic impairments, affecting naming through a different mechanism. Notably, such functional imaging findings are especially informative in the neuropsychological context of dissociations observed in Alzheimer's patients between semantic and phonological fluencies.^140^

In a similar manner for PNs, the lateral prefrontal cortex/the inferior frontal cortex may be engaged in the cognitive control aspects of lexical retrieval for PNs, as suggested by an fMRI study using a covert naming paradigm in older adults^92^ and by another one using an associative face/name learning task in healthy subjects.^141^ Lastly, the medial frontal regions, encompassing the orbitofrontal and anterior cingulate cortex, may be associated with the socio-emotional salience related to faces and could facilitate face naming.^26,28,29^

### Synthesis

We have previously shown that lexical retrieval processes primarily rely on temporal regions, although broader neural networks come into play to ensure naming efficiency. Consequently, naming impairments may arise from other causes than a pure lexical retrieval deficit. In line with this view, damage to various regions within the fronto-temporo-parietal regions can lead to naming failures.

## Summary and conclusion

At the cortical level, the retrieval processes associated with CNs and PNs appear to rely on a partially wide overlapping neural network, centred on temporal structures with extensions into frontal and temporo-parietal/inferior parietal regions (see Fig. 1 for a summary illustration of the cortical bases underpinning lexical access to CNs and PNs). Within these overlapping subnetworks, the BTLA and the ATS (especially the TP) emerge as pivotal components, likely assuming more specific roles for CN and PN retrieval, respectively. In agreement with this, evidence from VLSM studies conducted with glioma patients further showed that anomia for CNs resulted from damage to the mid-to-posterior ventral temporal cortex^44^ (see also^142,143^) whereas anomia for PNs is associated with damage to mid-to-anterior ventro-temporal structures.^42,43^

Thus, it could be that the brain specifically distinguishes, within a unified lexical access process, two grammatical classes due to additional cognitive demands required by PNs (i.e. unique psycholinguistic features, specific-level naming, greater memory resources, socio-emotional factors), compared to CNs. Nonetheless, it is not excluded that some CNs, especially those which are highly specific, atypical, unfamiliar, infrequent and/or very abstract, might be retrieved with the same neural resources as those used for PNs.

On the other hand, the ATS encompass an array of anatomical structures, each characterized by a unique connectional architecture to modality-specific cortical areas.^144^ Hence, it is conceivable that various subregions within the ATS may be associated with the processing of different conceptual/grammatical categories, in relation to distinct input modalities, and even distinct lexical/semantic processes. Indeed, the ATS have been the subject of multiple investigations, revealing a multi-faceted organization. In this sense, Rice *et al*.^145^ have notably demonstrated in an fMRI study that, beyond a localized ventral category/modality-invariant subregion, other anterior temporal areas are gradually specialized, based on person and social concepts. Accordingly, the dorsolateral and polar regions of the ATS may be specialized in some aspects of social cognition.^144,146-150^ Further, some studies have delineated a progressive auditory/visual division within the ATS, aligned along a superior to inferior axis.^149,151,152^ As a note of caution, it is important to recall that, before the methodological advances in fMRI data processing that enhanced signal quality in the ventral sectors of the ATS,^153^ these regions were particularly vulnerable to magnetic susceptibility artefacts, which could hinder the detection of their activation.^152,154,155^ This limitation should be taken into account when discussing older fMRI studies focusing on the ATS.

As a second note of caution, it should be emphasized that each neurological disease tends to affect only a specific set of brain regions. For instance, stroke injuries typically involve the territories of the middle cerebral and posterior cerebral arteries, while structures situated in the anterior temporal region or on the medial surface of the brain are rarely affected. Conversely, diseases such as semantic dementia or fronto-temporal dementia, which predominantly affect the ATS and the ventral prefrontal cortex, tend to spare the occipital, posterior temporal and parietal areas. In this context, lesion aetiologies and their distribution are crucial factors to consider when evaluating the various forms of evidence, particularly in relation to CN/PN retrieval. This also underscores the importance of conditions that frequently damage the ventral temporal cortex, especially glioma or temporal epilepsy surgery, as valuable sources of information for this topic.

In this initial section, we mainly discussed the involvement of cortical structures in lexical retrieval, which constitutes only a fraction of the narrative. A comprehensive understanding of this cognitive process at the broader cerebral scale necessitates a thorough investigation of white matter connectivity. This is of paramount significance in informing anatomical and neuro-computational models of language, particularly in unravelling the anatomical foundations of anomia, given that white matter connections are severely compromised across a large spectrum of neurological conditions.

## Exploring ventral connectivity: implications for proper name and common name retrieval

As comprehensively reviewed in the preceding sections, cortical areas within the ventral stream play a pivotal role in the process of lexical access. Our current focus shifts to delineating the white matter tracts that interconnect these cortical areas, the anatomical connectivity being increasingly recognized as vital building blocks for functional network integration.

**Figure 2:**
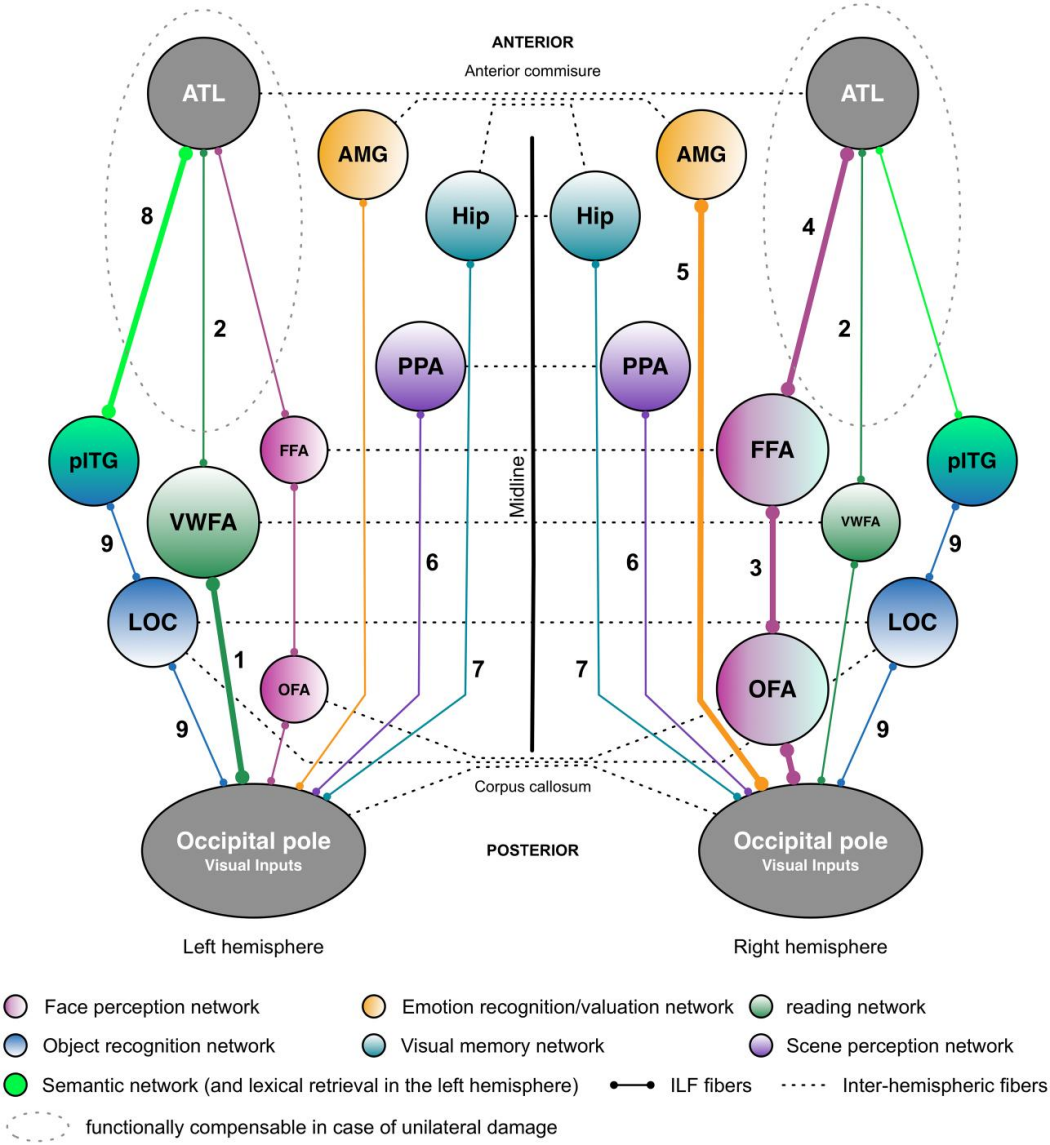
**Putative functional pathways supported by the multi-layered fibre organization of the ILF.** The summary illustration is derived from literature data regarding the ILF’s functions, primarily based on neuropsychological findings. Thicker lines and larger circles represent functional pathways biased towards a particular hemisphere. Neuropsychological syndromes resulting from ILF disruption include: (1) pure alexia; (2) written comprehension disorder; (3) apperceptive prosopagnosia; (4) associative prosopagnosia; (5) impairments in emotion recognition or hypo-emotionality; (6) scene perception impairments; (7) visual memory impairments; (8) semantic and/or lexical retrieval impairments; and (9) visual agnosia. AMG, amygdala; ATL, anterior temporal lobe; FFA, fusiform face area; Hip, hippocampus; LOC, lateral occipital cortex; OFA, occipital face area; pITG, posterior inferior temporal gyrus; PPA, parahippocampal place area; VWFA, visual word form area. The figure is reused from Herbet *et al*.^158^

**Figure 3:**
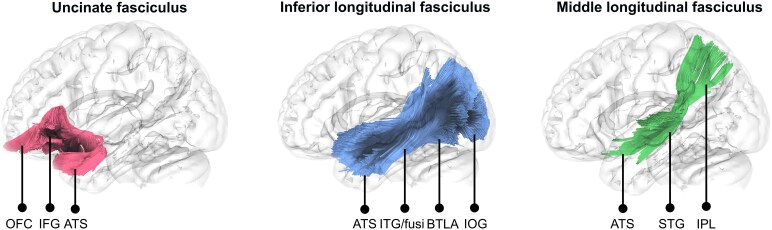
**Associative white matter tracts supporting lexical retrieval to CNs and/or PNs.** This figure highlights associative white matter tracts presumed to play a role in lexical retrieval for both CNs and PNs. The strength of evidence varies across the tracts: robust neuropsychological evidence supports the role of the ILF in picture-guided lexical retrieval for both CNs and PNs, while the role of the UF is more uncertain, with a potential implication in PN retrieval. The involvement of the MdLF in lexical retrieval remains hypothetical at this stage. The main cortical terminations of the tracts under scrutiny are indicated. ATS, anterior temporal structures; BTLA, basal temporal language area; Fusi, fusiform gyrus; IFG, inferior frontal gyrus; IOG, inferior occipital gyrus; IPL, inferior parietal lobe; ITG, inferior temporal gyrus; OFC, orbitofrontal cortex; STG, superior temporal gyrus.

From an anatomical perspective, the ILF runs longitudinally along the ventral aspect of the temporal lobe, originating from the occipital and occipito-temporal regions and terminating in various areas of the ATS.^156-161^ Recent investigations propose a complex, multi-layered fibre organization, encompassing three to four distinct branches, including fusiform, dorsolateral, lingual and potentially cuneal branches.^162,163^ In contrast, other studies suggest the presence of shorter connections, considered separate from the ILF (although this distinction is debatable), between various areas of the mid-to-posterior fusiform gyrus and the ATS.^164,165^ Consequently, the ILF is strategically well-positioned to establish connections between the mid-to-posterior ventral temporal regions (frequently associated with the BTLA) and the ATS. Thus, it appears to be a prime candidate for conveying lexical retrieval in this ventral neural network. Compelling empirical substantiation now firmly establishes that the left ILF plays a discernible role in both CN^44,45^ and PN retrieval,^42,43^ especially in visual modality, as expounded further below. Notably, this extended functional role is not mutually exclusive with its potential involvement in semantic processing.^161^ A schematic illustration of all functional implications attributed to the ILF is given in Fig. 2 and an overall view of the anatomical position and cortical terminations of the ILF is provided in Fig. 3.

## The role of the inferior longitudinal fasciculus in common name retrieval

In accordance with its anatomical course and cortical connections to areas crucial for lexical retrieval (Fig. 3), namely the mid/posterior lexical-semantic interface and, to a lesser extent, the ATS, there is a growing body of experimental evidence supporting the central role of the left ILF in the access to lexical representations. Thus, a large-scale neuropsychological study combining VLSM and tract-focused analyses was conducted with a sample of 110 patients having undergone surgical resection for a left low-grade glioma. In this study, it was revealed that anomia for CNs, while controlling for semantic disturbances (phonological errors being virtually absent), was linked to damage to the mid-to-posterior portion of the inferior temporal gyrus (extending to fusiform and middle temporal structures; see also^142,143^), in which the ILF is known to have cortical endpoints. Further and most importantly, the degree of residual tumour infiltration into the left ILF emerged as a robust predictor of this phenomenon.^44^

This observation aligns with the outcomes of a DES study, wherein it was unequivocally demonstrated that stimulation of the left ILF resulted in pure anomia for CNs, albeit only when the ATS remained free of tumoural infiltration.^45^ Conversely, in scenarios where the ATS were impacted by low-grade gliomas, the ILF ceased its typical function, possibly rerouting its role via alternative pathways,^45^ in accordance with the well-documented neuroplasticity potential of the ATS, especially in the pathophysiological context of slow-growing tumours.^16^ This overarching pattern of findings has recently been reaffirmed through a longitudinal multi-modal study that integrated DTI tractography and involved neurosurgical patients affected by a glioma.^48^ The authors of this study further showed that, prior to surgery, impaired performance in picture naming was primarily linked to damage of the mid-to-posterior part of the left ILF. Conversely, when the infiltration of the ILF did not result in a deficit, it was typically its anterior part that was damaged, often in association with the cortical ATS. Subsequent to surgery, naming impairments exhibited a robust correlation with the extent to which the ILF had been resected, albeit exclusively in cases where the ATS and the ILF had not been infiltrated prior to surgery. In the same way, it is noteworthy to mention the case reported by Herbet *et al*.,^45^ which detailed the experience of a single patient who developed severe and enduring anomic aphasia following surgical transection of the ILF (neither it nor the ATS were significantly infiltrated preoperatively), an intervention, necessitated to access a tumour located within the left temporo-mesial structures.

Another study, mapping the relationship between neurological lesions from diverse aetiologies and picture naming performance, has also consistently emphasized the causal role of the left ILF in lexical access to CNs (and not to PNs).^54^ Moreover, neuropsychological studies using tract-wise lesion analysis have unveiled causal links between the disruption of the left ILF and impaired object naming performance in individuals with brain tumours, either preoperatively^142^ or postoperatively.^143,166^ Additional indirect evidence is derived from studies, which have coupled DTI metrics with language performance assessments, across various patient populations exhibiting difficulties in naming. These populations include individuals with primary progressive aphasia (logopenic variant),^167^ stroke^168,169^ and temporal epilepsy or epilepsy-related temporal surgery.^170^ In general, these studies have identified non-specific associations between ILF DTI metrics (primarily fractional anisotropy)^167-170^ and object naming performance.

### Synthesis

In short, the existing literature highlights the central role of the left ILF in object naming, likely related to the process of CN retrieval. Notably, the most direct and compelling evidence originates from studies involving patients with brain tumours. This is unsurprising given that stroke injuries typically have a limited impact on ventro-temporal structures, in contrast to lower-grade gliomas. Interestingly, studies involving glioma patients have further shown that the lesion of the ILF can be compensated for in lexical retrieval but uniquely when its anterior part is infiltrated along with the ATS, suggesting the recruitment of specific brain plasticity mechanisms. Finally, current evidence regarding the implication of other ventral tracts in lexical retrieval remains limited. In the following section, we will demonstrate how the ILF implication extends to PNs.

## The role of the uncinate and inferior longitudinal fasciculi in proper name retrieval

The available data concerning the connectional anatomy of PN retrieval is limited. Seminal work in this regard was initially presented by Papagno *et al*.^46^ over a decade ago, and subsequently complemented by additional neuropsychological investigations,^54,171^ including ours.^42,43^ We delve into these reports below.

### The uncinate fasciculus

Employing a longitudinal experimental design involving patients undergoing surgical resections for gliomas, Papagno *et al*.^46^ observed a decline in PN retrieval performance, as evaluated through a FFN task, after the removal of the left UF. This C-shaped fronto-temporal tract interconnects the ATS with orbital, ventro-lateral and anterior frontal regions^156,159,172-174^ (for a review, see Von der Heide *et al*.^175^) via a multi-layer fiber organization (see Fig. 3).^172,173^ Importantly, in Papagno *et al*.’s^46^ study, the impairment in FFN was more pronounced when the temporal part of the UF was affected, compared to its frontal part. Further analyses indicated that, in patients with UF resection, anomia for PNs did not fully recover, even 9 to 12 months post-surgery (in contrast to anomia for CNs).^47^ Furthermore, a large-scale lesion-symptom study involving predominantly stroke patients demonstrated that anomia for PNs was predicted by the extent of damage to the left UF (and not to the ILF).^54^ In a more anecdotal context, a progressive decline in PN retrieval ability (but not in CN retrieval) was observed during the surgical resection of the left UF in two patients operated on while awake for temporal gliomas, and in a third patient during the UF stimulation, using stereo-electroencephalography.^171^

### The inferior longitudinal fasciculus

The involvement of the left UF in PN retrieval has been called into question by two recent studies involving patients with lower-grade gliomas.^42^  ^,43^ In the first study, a retrospective assessment was conducted in 58 patients in the chronic post-surgical phase (i.e. at least 3 months after surgery) to evaluate their ability to retrieve PNs from the mental lexicon, utilizing a FFN task.^43^ Performance was analysed through a multi-modal approach that included location-based between-group analyses of resection areas, VLSM and disconnection-symptom mapping. These comprehensive analyses also considered various factors influencing FFN performance. Specifically, sociodemographic variables (i.e. age and educational attainment) were included in ‘Model 1’, associated with FFN task-related control factors (i.e. self-estimated familiarity with each seen face and biographical knowledge) in ‘Model 2’ or with interrelated cognitive processes (non-verbal semantic processing, verbal episodic memory and CN retrieval) in ‘Model 3’ while all factors were combined in ‘Model 4’. In summary, the patient group showed significantly poorer performance compared to a group of 72 matched healthy participants. VLSM results revealed that damage to the mid-to-anterior part of the inferolateral and ventro-basal temporal cortex (including the TP) was particularly predictive of impaired PN retrieval. Most notably, surgery-related disruption of the left ILF was robustly associated with FFN scores, as indicated by disconnection-symptom mapping (i.e. tract-by-tract correlation analyses and spatial correlations), across all tested models. In a separate double-case study, this research^43^ finally demonstrated that the interruption of the right ILF in a left-handed patient resulted in a severe and long-lasting PN retrieval deficit, while the same interruption in a right-handed patient did not cause such an impairment. This finding showed that damage to the right ILF can lead to crossed anomia for PNs in left-handed individuals, reinforcing the main conclusions of this study.

It is noteworthy that the left UF did not emerge as a contributor to PN retrieval impairment, in this work.^43^ Specifically, the resection location-based between-group analyses conducted in this study indicated that anomia for PNs could occur following damage to the ILF while sparing the UF entirely. Indeed, patients with surgical resections centered on the ATS extending to neighbouring temporal structures (i.e. both UF and ILF damaged) as well as those with resections in the mid-to-posterior inferior temporal gyrus (i.e. ILF exclusively damaged) had significantly lower FFN scores than patients who underwent resections in the dorsal frontal areas (i.e. where both ILF and UF were fully spared) or in the fronto-temporo-insular areas (i.e. with extensive UF damage and sparsely, minimal ILF damage) but no other differences have been observed between these groups.

In the second study, Burkhardt *et al*.^42^ presented three patient cases, all affected by a low-grade glioma involving the mid-to-anterior portion of the temporal lobe. Employing the same FFN task as in the previous study,^43^ a longitudinal assessment was conducted by the first author (E.B.) in these patients, at three time-points: 1 day pre-surgery, 4 days post-surgery and 3 months post-surgery. This evaluation was complemented by a comprehensive neuropsychological examination. Importantly, all patients exhibited an enduring decline in PN retrieval performance following the surgical procedure. This decline could not be attributed to a lack of familiarity with famous faces nor to insufficient biographical knowledge. Of note, only one patient, B.S., continued to experience persisting difficulties in retrieving CNs, thereby underscoring the specificity of the PN retrieval deficit in the two remaining patients, A.H. and C.K., in the context of their lexical access abilities. Notably, the neuropsychological examination of A.H. remained completely free for difficulties in other cognitive functions assessed, particularly verbal episodic memory. From an anatomical perspective, detailed disconnection analyses revealed that the ILF was the only white matter tract consistently disrupted across all three patients. Specifically, these analyses pinpointed the ventrolateral segment of the ILF, projecting into the implicated anterior ventrolateral temporal cortex.

### Contrasting the involvement of the inferior longitudinal fasciculus and the uncinate fasciculus

The two aforementioned studies represent a notable leap forward in the understanding of the connectional anatomy underpinning lexical access to PNs, at least for famous faces. The proposition of the central role of the ILF stands in contrast to prior reports, which had rather suggested the involvement of the left UF.^46,54^ This discrepancy in findings may be attributed to differences in methodological approaches. The studies by Papagno *et al*.^46^ and Mehta *et al*.^54^ did not consider the interplay of various cognitive processes, which may be involved in PN retrieval. Consequently, the correlations observed between UF damage and PN retrieval deficits in these studies may also reflect the influence of other participating processes.

Indeed, the integrity of the UF has been associated with various cognitive domains, including semantic processing^168,176,177^ and episodic memory.^178^  ^,179^ In this sense, some findings indicate that the microstructural properties of this tract correlate with associative learning performance, notably in tasks involving face-name pairing.^180,181^ In this respect, Alm *et al*.^180^ suggested that the UF implication in associative learning tasks may be related to its role in episodic memory, potentially under stringent conditions of retrieval competition. In this context, the faces and names employed in their experimental task were indeed designed to be closely similar and frequent respectively, in order to increase the difficulty of distinguishing between possible retrieval candidates.^180^ This possible implication of the UF seems broadly consistent with its projections in the inferior frontal cortex, whose role in the executive component of selecting the lexical-semantic target among concurrent alternatives^138,141,182^ is well known. Notably, competition is likely minimal in the context of retrieval labels relatively unique, necessarily specific and rather infrequent compared to CNs, named at a basic level. However, the integrity of such a control mechanism might be critical in PN retrieval, taking into account the highly precise and individualized word selection process required within a homogeneous perceptual-semantic contexts. On the other hand, several works proposed a role for the left UF in socio-emotional functions,^144,175^ including emotional valence-based mnemonic associations,^175^ possibly oriented towards face-name processing. Once again, that seems congruent with its cortical terminations in both the ATS (implicated in mnemonic association of person-related information)^175^ and the face-selective region of the orbitofrontal cortex, which is implicated in social motivation processing.^183^

Upon closer examination, it is likewise essential to acknowledge that the study by Papagno *et al*. did not experimentally investigate the involvement of other anatomical connections, notably the ILF.^46,47^ In contrast, anatomical analyses in Burkhardt *et al*.^43^ found an implication of the left ILF controlled for the implication of other tracts, including the UF. Furthermore, the ILF was the unique tract damaged in common across the three patients, in Burkhardt *et al*.^42^ Lastly, it is worth noting that in the study conducted by Mehta *et al*.,^54^ the ILF primarily incurred damage to its anterior segment. This was attributable to the characteristics of the patient population, consisting mainly of cerebrovascular lesions and less frequently anterior temporal lobectomies for epilepsy. Disentangling the distinct contributions of the ILF and the UF in this context becomes exceedingly challenging, as both tracts terminate in the ATS (for the ILF^163^; for the UF^173^). This challenge is also evident in the study by Koay *et al*.,^171^ where PN retrieval deficits following stimulation of the white matter within the ATS were attributed to a virtual lesion of the left UF.

### Synthesis

To date, only a handful of studies have examined the anatomical connectivity underlying PN retrieval. Some neuropsychological findings have suggested that the left UF may play a critical role in this process. However, more recent evidence instead highlighted the central role of the left ILF, not that of the UF. Several methodological considerations could explain these divergent results. Nonetheless, the processes potentially linking the UF to the retrieval of PNs have been discussed.

## Summary and conclusion

Taken as a whole, the most up-to-date literature corroborates the central role of the left ILF in both CN and PN retrieval processes, contrary to that of the UF. It aligns with the cortical implications discussed in the first part of this review, particularly the finding that the posterior and anterior ventral temporal cortex are predominantly involved in CN and PN retrieval, respectively. Indeed, the ILF is known to project into both of these regions, forming a coherent cortico-subcortical system for lexical retrieval with distinct specificities. However, it is important to emphasize that clearly dissociating the precise contributions of the cortex versus white matter tracts remains highly challenging, as both are often simultaneously and indistinguishably affected in most neurological conditions. Furthermore, standard lesion-symptom mapping methodologies are still suboptimal for accurately delineating the pathological variances associated with each type of structure. In this context, DES of white matter tracts represents a promising approach to dissociate the contributions of ventral tracts to the retrieval of CNs and PNs. This direction is both scientifically and clinically justified, as evidence suggests that patients undergoing inferior temporal regions resection along with ILF damage, do not recover from aphasic anomia,^44^ significantly impacting their quality of life. In brief, there is a clear need for well-designed and adequately powered studies to further discern the precise functional implications of the ILF fibres in naming, especially from the cortical contributions.^184^

In the final section of this review, we present hypothetical models outlining dissociated patterns of ventral connections, which underlie CN and PN retrieval respectively, seeking to elucidate the complex network of regions and pathways contributing to these essential language processes. Thus, we propose several promising avenues for future research, aiming to further refine our comprehension of the neural mechanisms governing naming processes, depending on input modality and/or semantic category (at least for PNs for this latter).

## Hypothetical white matter architecture supporting lexical access to common names and proper names

### Separate pathways for common name and proper name retrieval?

In light of the increasingly established role of the left ILF in both CN^44,48^ and PN^42,43^ retrieval, a significant question arises: do separate white matter components within the ILF serve to transmit psycholinguistic information necessary for naming entities belonging to each of these grammatical categories? This hypothesis gains relevance in the context of reports highlighting dissociations between anomia for CNs and PNs resulting from various brain lesions,^25,26^ and of findings from VLSM studies that have precisely located the critical cortical locus for PN retrieval as being more anterior along the ventrolateral temporal axis,^43^ compared to CN retrieval.^44^ To substantiate this hypothesis, empirical evidence is imperative, demonstrating that well-defined, selective damage to the left ILF yields either anomia for CNs or anomia for PNs, whereas more extensive lesions result in a shared lexical retrieval deficit. In support of this, recent research has providedconsistent evidence showing that damage to the ventrolateral fibres of the ILF along with the antero-ventral temporal cortex, led to pure anomia for PNs without concurrent anomia for CNs (at least as assessed by a standard picture-based object naming task), in two out of the three patients studied.^42^ Notably, the third patient, who exhibited anomia for both CNs and PNs, had the largest lesion.

### Segregated fibres for semantic categories-dependent proper name retrieval?

As widely discussed above, previous studies have strongly implicated the ILF in the retrieval network of FFN.^42,43^ Remarkably, these findings parallel the involvement of the right ILF in face recognition/face memory, as demonstrated in both healthy individuals^185-188^ and prosopagnosic patients^189,190^ (see also^191^ for a comprehensive review). In this context, it is essential to consider whether ILF’s involvement in PN retrieval is specific to certain semantic categories, possibly limited to faces. Against it, existing research has already associated the ATS—in which the rostral fibres of the ILF project—with landmark name retrieval,^62,114^ extending their role beyond the processing of person-specific information,^192^ as part of the ‘extended system’ for face stimuli.^193^ Moreover, considering the specialization of the fusiform and parahippocampal regions for face^193-196^ and spatial/place (including landmarks, scenes and topographical features) processing^197-199^ respectively, the hypothesis that distinct components of the left ILF may be selectively involved in PN retrieval, depending on the semantic category, becomes especially relevant. Indeed, given its caudal projections into the mid-to-posterior fusiform and parahippocampal structures, the ILF’s role in face (for the former) and landmark (for the latter) name retrieval, warrants further investigation. Along these lines, recent studies have identified, in the right hemisphere, distinct white matter pathways that interconnect these face- and place-selective areas with the ATS^164,200^ (see also^165^ for face areas), and whose microstructural properties have been associated with recognition performance for each stimulus type.^164^ It is therefore plausible that similarly segregated connections within the left ventral temporal structures play a role in the retrieval of face names and landmark names, respectively.

### A putative role of the middle longitudinal fasciculus in auditory-based lexical retrieval?

As comprehensively reviewed by Herbet *et al*.^158^ and expounded upon by Zemmoura *et al*.,^161^ the ILF represents a central white matter tract involved in cognitive and behavioural processes, grounded in the visual modality. This alignment between the tract’s connective properties and its functional implications is evident in its projections to associative visual areas and its involvement in tasks such as reading, visual recognition as well as lexical access, based on object pictures^44,45^ and famous faces.^42^  ^,43^ Currently, there is no conclusive evidence that the ILF mediates lexical retrieval when processing information in the auditory modality. While a few isolated studies have all the same hinted at its potential involvement in certain auditory-derived functions, such as naming from aural descriptions^170^ or auditory-verbal comprehension,^161^ this remains an area in need of further investigation.

From an anatomical viewpoint, the middle longitudinal fasciculus (MdLF) seems better placed to convey the auditory-linguistic information critical for efficient lexical retrievalof CNs and PNs. Through a multi-layered organization,^201-203^ the MdLF interconnects the ATS with parietal and occipital regions. More specifically, it passes through the superior temporal structures/transverse gyri, projecting rostrally into the anterior part of the superior temporal gyrus (particularly in the dorsal TP) and posteriorly into the inferior and superior parietal lobules, as well as occipito-parietal and occipital areas.^201-208^ Notably, it has terminations in the primary and associative auditory cortex.^201,202^ The MdLF is displayed in Fig. 3.

In view of its connectivity pattern, it has been hypothesized to play a role in sound and auditory language processing.^209^ It might also contribute to coding sublexical representations into articulatory forms, to supporting aspects of acoustic-phonetic word processing^210^ and acting as an interface between auditory representation and semantic/lexical access.^201^ Nevertheless, the precise connective features—including components, cortical projections, and interindividual variability—of the MdLF have not been fully determined, and studies assessing its possible role in aspects of language processing are limited.^211^

One electrostimulation mapping study investigated the potential implication of the MdLF in language processing in eight patients undergoing neurosurgery for low-grade gliomas.^212^ No language interference was observed after stimulation of the left MdLF and no permanent deficits were found after surgical resection of its anterior part. Nonetheless, it does not inform about the potential implication of its posterior part. Additionally, the assessment of naming was only performed in the visual modality (through picture naming), which does not rule out a potential role of the MdLF in auditory naming (based on verbal descriptions, sounds, or voices).

Otherwise, the MdLF and the ILF share a close anatomical relationship, characterized by their projections into the TP and a parallel course along the temporal lobe and then into the occipital lobe.^201,202^ However, the ILF follows a more ventral and lateral trajectory compared to the MdLF. Consequently, its anterior projections target the ventral TP, while those of the MdLF terminate in the dorsal TP (Fig. 3).^201-204^ This spatial arrangement highlights the possible distinct roles of the ILF and the MdLF in mediating visual and auditory language processing, respectively.^56^

As a point of emphasis, beyond the transmodal role of the left ATS in PN retrieval/naming people from either faces or voices,^60,61^ some of their sectors are thought to consist of distinct modality-dependent regions responsive to auditory, visual and audiovisual stimuli in the dorsal, ventral, and polar parts, respectively.^149^ This aligns, in a certain way, with the connectivity-constrained ‘hub-and-spoke’ theory, which posits that modality-specific semantic information is channelled through spokes within the graded transmodal ATS.^110^ Consequently, visual and auditory semantic processing may proceed independently within the inferior and superior temporal gyri, before converging within the ATS, through a graded integration.^56,152^ In the context of naming unique entities, electrophysiological evidence indeed indicated that surrounding and more posterior modality-dependent areas within the ventro-basal temporal cortex and the superior temporal gyrus, exhibit selective responses to faces and voices, respectively.^61^ Interestingly, this finding mirrors the neuropsychological evidence regarding person recognition disorders from faces and voices, associated to the right hemisphere.^213^ Overall, these functional implications suggest dissociated roles for the left ILF and MdLF in lexical(-semantic) processing, depending on the input modality.

In summary, the results presented here suggest that the left MdLF may play a role in integrating psycholinguistic-related information from the auditory modality into the ATS, thereby contributing to auditory-based lexical retrieval, whether for PNs or CNs. Damage to the left MdLF might thus result in an auditory modality-specific anomia, potentially selective to voices. This neuropsychological impairment, known as ‘phonoanomia’, has notably been described in a recent case study by Moretta and Trojano.^214^

### Summary and conclusion

In this section, we proposed the existence of distinct ILF fibre pathways dedicated to CN and PN retrieval, respectively. We then posited that, in the context of PN retrieval, specific components of the left ILF may be selectively involved in retrieving face names and landmark names, given its caudal terminations in cortical areas specialized for these perceptual-conceptual categories. Finally, we hypothesized that the MdLF might contribute to auditory-guided naming, particularly in lexical retrieval of CNs and PNs. This final section allowed us to elaborate on several anatomical hypotheses regarding the neural organization of white matter pathways underpinning lexical access, based on grammatical and semantic categories and depending on the input modality. The ILF, MdLF (and UF) are represented in Fig. 3.

## General conclusion

In this article, we conducted a comprehensive review of the brain network responsible for CN and PN retrieval, shedding light on the cognitive processes that distinguish these two grammatical categories. While CN and PN retrieval exhibit psycholinguistic differences, primarily in terms of mapping relationships between words and their referents (i.e. a one-to-one mapping between meaningless PNs and their unique associated entities versus a one-to-many mapping between meaningful CNs and the category-level objects named), we highlighted that an overlapping cortical network, situated along the ventral language stream, supports lexical access to both CNs and PNs. Overall, this network encompasses fronto-temporo-parietal regions, though each area may not hold equal importance in the retrieval of the two grammatical categories. In this sense, the BTLA (i.e. the mid-to-posterior part of the ventral temporal structures) and the ATS, two pivotal functional hubs within this network, appear to play relatively greater roles in CN retrieval for the former, and in PN retrieval for the latter.

At the structural connectivity level, our review emphasized the critical role of the left ILF in visually guided lexical access to CNs and PNs. Disruption of this white matter tract thus leads to a persistent anomia for both grammatical categories. Recent findings have especially highlighted the ILF’s paramount role in PN retrieval and challenged the long-held hypothesis of the left UF, at least as the main neural pathway implicated in PN retrieval. In light of these new findings, we proposed several anatomical hypotheses regarding the connectional architecture underlying PN and CN retrieval. These hypotheses aim to stimulate and guide empirical investigations, so future research can deepen our understanding of the complex neural mechanisms governing lexical access.

In brief, our review underscores the ILF's implication in lexical access to CNs and PNs in the visual modality, supporting an overlapping cortical network, likely centred around different functional epicentres along the temporal structures. In this way, our review significantly updates the dual-pathway model for language.^6,11,15^ Furthermore, by proposing that disruption of distinct anatomical connectional networks may entail anomia for CNs or PNs —taking into account PN subcategories and input modality irrespective of the name's grammatical class— our review opens promising avenues for future research. Our hypotheses indeed aim to stimulate and guide empirical investigations in order to enrich the understanding of the complex neural mechanisms governing lexical access.

Beyond these fundamental insights, significant clinical applications are anticipated. Indeed, they contribute to improved practices in awake surgeries for brain tumours and may support functional mapping in epilepsy, enabling better identification and preservation of eloquent structures involved in PN and CN retrieval. More generally, our review paves the way for a better understanding of neuropsycholinguistic profiles of patients, affected by various neurological pathologies targeting the temporal lobe, such as epilepsy, tumours and related surgeries but also with dementia, strokes, and others. Finally, these findings have implications for language rehabilitation, as many neurologically related language disorders are predominantly associated with white matter connectivity damage, not only in patients undergoing brain tumour surgery but also in those recovering from strokes, the most common cause of aphasia.^215^ Hence, identifying white matter lesions associated with anomia, in relation to the grammatical category and the input modality, may represent a promising avenue for refining clinical care related to speech-language therapy.

## Data Availability

Data sharing is not applicable to this article as no new data were created or analysed in this study.
